# Investigation of a linezolid-resistant *Staphylococcus epidermidis* outbreak in a French hospital: phenotypic, genotypic, and clinical characterization

**DOI:** 10.3389/fmicb.2024.1455945

**Published:** 2024-09-11

**Authors:** Nadège Lépine, José Bras-Cachinho, Eva Couratin, Coralie Lemaire, Laura Chaufour, Armelle Junchat, Marie-Frédérique Lartigue

**Affiliations:** ^1^Service de Bactériologie-Virologie-Hygiène, Centre Hospitalier Universitaire de Tours, Tours, France; ^2^ISP, UMR1282, Université de Tours, INRAe, Tours, France; ^3^Equipe Opérationnelle d'Hygiène, Centre Hospitalier Universitaire de Tours, Tours, France

**Keywords:** *Staphylococcus epidermidis*, linezolid, multi-drug resistance, genotyping, sequence type ST2, IR-Biotyper^®^

## Abstract

**Purpose:**

We aimed to retrospectively investigate an outbreak of linezolid-resistant *Staphylococcus epidermidis* (LRSE), at Tours University Hospital between 2017 and 2021.

**Methods:**

Twenty of the 34 LRSE isolates were included in the study. Antimicrobial susceptibility testing was performed using the disk diffusion method and MICs of last-resort antibiotics were determined using broth microdilution or Etest^®^. Seventeen of the 20 resistant strains were sent to the French National Reference Centre for *Staphylococci* to determine the mechanism of resistance to linezolid. The clonal relationship between LRSE strains was assessed by PFGE and the sequence type determined by MLST. We retrospectively evaluated a new typing tool, IR-Biotyper^®^, and compared its results to PFGE to evaluate its relevance for *S. epidermidis* typing. Medical records were reviewed, and antibiotic consumption was determined. Search for a cross transmission was performed.

**Results:**

All LRSE strains showed high levels of resistance to linezolid (MICs ≥ 256 mg/L) and were multi-drug resistant. Linezolid resistance was associated with the 23S rRNA G2576T mutation and none of the 17 strains analyzed carried the *cfr* gene. Ninety-five percent of the 20 LRSE studied strains were genetically related and belonged to sequence-type ST2. The dendrogram obtained from IR-Biotyper^®^ showed 87% congruence with the PFGE analysis. Prior to isolation of the LRSE strain, 70% of patients received linezolid. No patients stayed successively in the same room.

**Conclusion:**

Linezolid exposure may promote the survival and spread of LRSE strains. At Tours University Hospital, acquisition of the resistant clone may also have been triggered by hand-to-hand transmission by healthcare workers. In addition, IR-Biotyper^®^ is a promising typing tool for the study of clonal outbreaks due to its low cost and short turnaround time, although further studies are needed to assess the optimal analytical parameters for routine use.

## 1 Introduction

Coagulase-negative *Staphylococci* (CoNS), including *Staphylococcus epidermidis*, are opportunistic pathogens that are frequently implicated in human disease, particularly in the context of healthcare-associated infections such as catheter-associated bloodstream or prosthetic joint infections (PJI) in immunocompromised patients. Linezolid is one of the most widely used antibiotics against Gram-positive cocci. Linezolid resistance is mediated by different mechanisms, often co-expressed. Mutations in 23S rRNA at linezolid binding sites (the G2576U substitution being the most frequent) and mutations in ribosomal proteins L3 and L4 located on the surface of the 50S RNA subunit are the most prevalent. Acquisition of plasmid-mediated multidrug resistance genes, either *cfr* encoding an RNA-methyltransferase, or *optrA* or *poxtA* encoding ABC transporters, confers transferable resistance to oxazolidinones (Brenciani et al., 2022; Long and Vester, 2012). Global surveillance studies report that only 0.75% of CoNS are resistant to linezolid (Flamm et al., 2016). Nevertheless, the spread of LRSE is an emerging public health concern because methicillin and linezolid resistance are often combined, leaving very few therapeutic options.

Numerous previous studies have already reported hospital outbreaks of linezolid-resistant *S. epidermidis* (LRSE) from countries around the world (Bouiller et al., 2020; Dortet et al., 2018; Kelly et al., 2008; Liakopoulos et al., 2010). Coustillères et al. described highly linezolid-resistant *S. epidermidis* strains isolated from patients with PJI since the introduction of protocolized postoperative linezolid in six French referral centers, including Tours University Hospital. They noted that LRSE carriage appeared to be directly related to linezolid use (Coustillères et al., 2023).

An increase in the incidence of LRSE has however been reported despite a decrease in linezolid use in some institutions (Huber et al., 2021). Linezolid resistance has also been observed in patients with no history of linezolid administration (Huber et al., 2021; Kelly et al., 2008; Liakopoulos et al., 2010; Seral et al., 2011), suggesting that clonal cross-transmission may lead to the emergence of LRSE outbreaks in addition to the selection of resistant strains under linezolid antibiotic treatment (Mihaila et al., 2012).

In this study, we report an outbreak of LRSE mainly in the orthopedic surgery department and the surgical intensive care unit (ICU) of the Tours University Hospital between 2017 and 2021. The strains were phenotypically and genotypically characterized to demonstrate their clonal relationship. These results were linked with the patients’ clinical characteristics in order to analyze potential risk factors for the acquisition of clonal LRSE strains. Furthermore, the ability of IR-Biotyper^®^ (IRBT) as a *S. epidermidis* typing method was evaluated and these results were compared to those of pulsed-field gel electrophoresis (PFGE), given the lack of bibliographic data on *S. epidermidis* IRBT typing.

## 2 Materials and methods

### 2.1 Bacterial strains

Thirty-four LRSE strains were isolated from clinical diagnostic samples between 2017 and 2021 at the Tours University Hospital. However, only 20 isolates were included as the other 14 were not preserved due to the retrospective nature of the study. In addition, linezolid-susceptible *S. epidermidis* (LSSE) strains had been isolated from three patients before their LRSE infection. These three isolates were also included in the study.

In accordance with standard hospital laboratory methods, strains were previously identified to species level by matrix-assisted laser desorption/ionization time-of-flight mass spectrometry (MALDI-TOF MS, Bruker Daltonik, Bremen, Germany) using the Biotyper reference library. Linezolid resistance was detected by the disk diffusion method (30 μg) and/or E-test^®^. The isolates were then stored appropriately at −80°C. All subsequent analyses were carried out on fresh strains after 18 to 24 h of aerobic incubation on Columbia blood agar plates at 35 ± 2°C.

A random isolate of *S. epidermidis* was used as an unrelated PFGE control strain. It was susceptible to methicillin and linezolid.

### 2.2 Susceptibility testing

*In vitro* phenotypic antimicrobial susceptibility testing was performed using the disk diffusion method according to CASFM-EUCAST 2021 recommendations for penicillin G, kanamycin, tobramycin, gentamicin, ofloxacin, tetracycline, rifampicin, and trimethoprim-sulfamethoxazole. Methicillin-resistance was detected with cefoxitin disks (30 μg). The following MICs were determined using the E-test method: ceftaroline, ceftobiprole, linezolid, tedizolid, daptomycin, dalbavancin, tigecycline, and delafloxacin. Susceptibility to vancomycin and teicoplanin was determined by the broth microdilution method. The results were interpreted using the CASFM-EUCAST 2021 *Staphylococcus aureus* breakpoints.

Seventeen of the 20 LRSE strains were sent to the French National Reference Centre for *Staphylococci*: genetic determinants of resistance were evaluated by whole genome sequencing. The acquired *mecA* gene, the acquired *cfr, optrA* and *poxtA* resistance genes, as well as point mutations in 23S rRNA were investigated by *in silico* analysis (Côrtes et al., 2022).

### 2.3 Patient characteristics

Medical record review was used to retrospectively collect demographic and clinical data. Search for a cross transmission was performed. Written information about the study was posted in each center and the non-opposition of each patient was sought before inclusion. Ethic approval was not required.

### 2.4 Linezolid usage data

Annual data on linezolid use were examined in the surgical ICU and orthopedic surgery from 2017 to 2021. Linezolid use was measured in Defined Daily Doses (DDDs) per 100 patient-days. Doses of 1,200 mg/day were considered as 1 DDD. These data were compared with the number of LRSE strains in these two units. The aim was to identify whether there was a trend in the use of linezolid and whether this might be linked to the emergence of linezolid resistance in *S. epidermidis* at Tours University Hospital.

### 2.5 PFGE and MLST typing

To search for clonality, strains were genotyped by pulsed-field gel electrophoresis (PFGE) of *Sma*I-digested total DNA for molecular typing, as previously described (Neoh et al., 2019), allowing bacterial isolates to be clustered into pulsotypes. We further characterized our strains using multilocus sequence typing (MLST) (Thomas et al., 2007) using primers listed in Supplementary Table S1. We assigned sequence types (STs) to each allelic profile using an MLST website.^1^

### 2.6 FTIR spectroscopic analysis

Further phenotyping studies were performed using a new typing method, the IR-Biotyper^®^ (IRBT) (Bruker Daltonik, Bremen, Germany), a Fourier transform infrared (FTIR) spectroscopy system that provides cost-effective results within 4 h (Hong et al., 2022).

**Figure 1:**
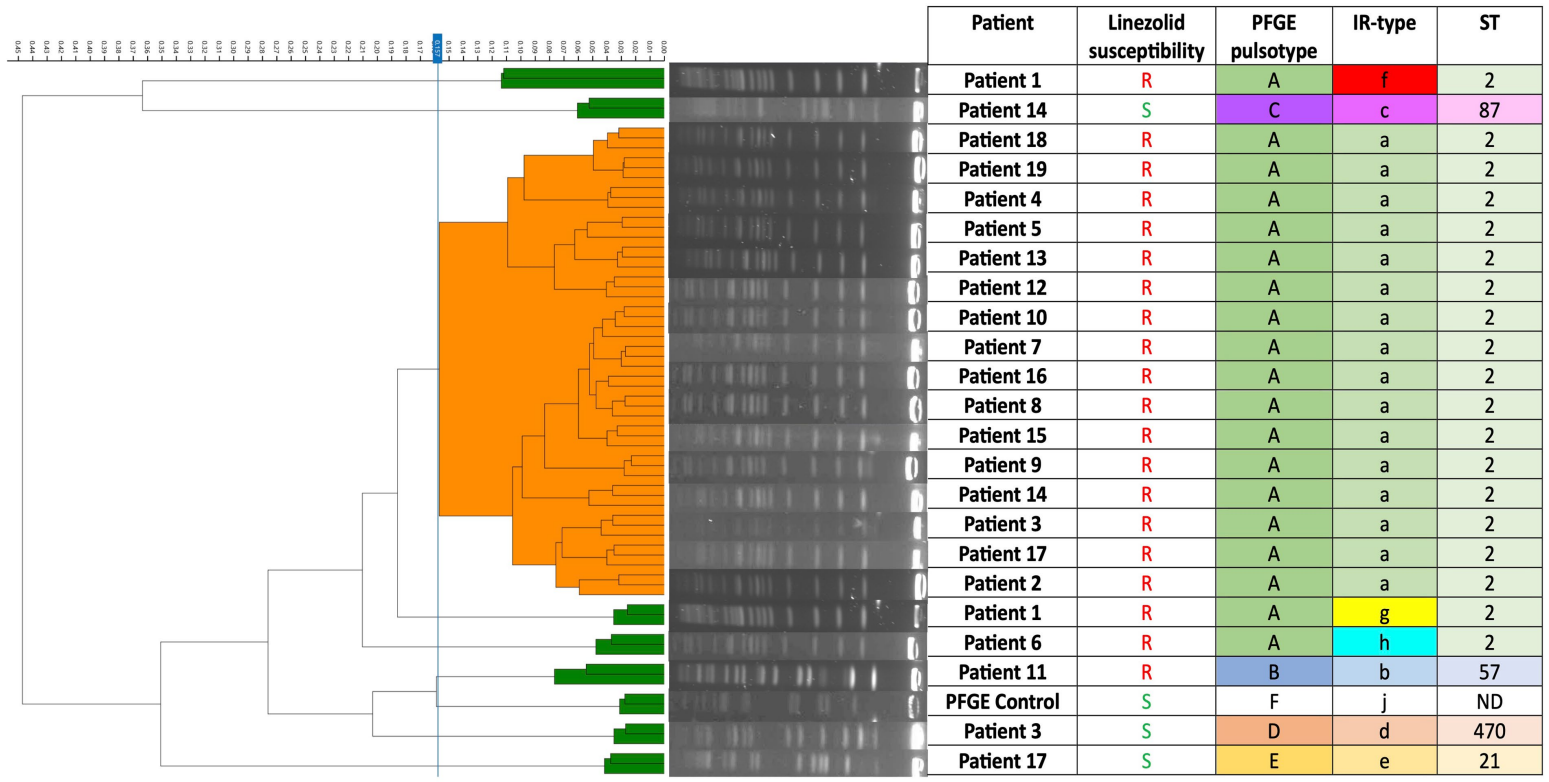
Dendrogram of 24 *Staphylococcus epidermidis* isolates (including one unrelated control strain) based on IR-Biotyper^®^ spectra. In the dendrogram, isolates that were clustered using the IR-Biotyper^®^ are shown in orange, while those that were not clustered are shown in green. ND, No Data.

Here, we retrospectively tested 22 of the 23 isolates from our study after they had been genotyped by PFGE. Each sample was analyzed in triplicate in a single experiment (Figure 1). Ethanol/water suspensions were prepared from blood agar cultures according to the manufacturer’s recommendations. We applied each bacterial suspension in triplicate to the FTIR silicon plate along with the two quality control spots provided in the kit. Dendrograms were automatically generated after spectra acquisition. The IRBT software automatically calculates a cut-off value that defines the distance at which spectra are considered to belong to the same cluster.

## 3 Results

### 3.1 Patient characteristics

In this study, 20 patients were retrospectively included with at least one LRSE isolate. Fourteen were male and six were female patients, with a mean age of 65 ± 16 years. These patients were hospitalized in three clinical departments: orthopedic surgery (12 patients), surgical ICU (6 patients), and gastrointestinal and liver surgery (2 patients). The clinical characteristics of the patients are shown in Table 1. The majority had at least one comorbidity, including type 2 diabetes (30%), hypertension (45%), or obesity (35%).

**Table 1:** Clinical characteristics of patients.

Patient	Ward of occurrence	Period of hospitalization (days)	Age	M/F	Initial BMI	Cause of admission	Date of culture	Culture site	No of LRSE positive samples	Prior LSSE isolation	Prior exposure to linezolid within 3 months Y/N (days)	Other antimicrobial treatment prior LRSE isolation	LRSE triggered antibiotic therapy (Y/N, molecule)	Infection, colonization, or contamination
1	Digestive and liver surgery	06/08/2017 to 16/10/2017 (71)	64	M	25.3	Liver transplant	19/08/2017	Peritoneal fluid	1/2	N	ND	ND	ND	ND
2	Digestive and liver surgery	11/07/2018 to 09/11/2018 (121)	61	F	23.7	Liver transplant	28/09/2018	Ascites	1/3	N	Y (14)	Ertapenem Ceftriaxone Ofloxacin PTZ Ceftazidime-avibactam	N	Colonization or contamination
3	Orthopedic surgery	04/11/2018 to 13/11/2018 (9)	61	M	26.5	Two-stage shoulder prosthesis exchange	05/11/2018	Right shoulder biopsy	3/5	Y: 1/5 MSSE (06/08/2018)	N	Levofloxacin Rifampicin	Y: doxycycline	Infection
4	Surgical ICU	06/01/2019 to 24/04/2019 (108)	50	M	25	Acute pancreatitis	30/01/2019	Peritoneal fluid	3/3 (Enriched broth culture only)	N	N	Ceftriaxone Metronidazole Amikacin Ciprofloxacin Ceftazidime PTZ Meropenem Vancomycin Cefepime	Y: vancomycin	Infection
5	Surgical ICU	28/02/2019 to 19/03/2019 (19)	74	M	31.5	Liver abscess, peritonitis, septic shock	28/02/201904/03/201910/03/2019	BAL Hepatectomy Blood	1 (10^6^LRSE) 2/5 1/4 bottle	N	Y (5)	PTZ Vancomycin Ceftriaxone Metronidazole Cefepime Ciprofloxacin	Y: vancomycin	Colonization
6	Orthopedic surgery	24/06/2019 to 05/07/2019 (11)	73	M	34.8	Two-stage hip prosthesis exchange	27/06/2019	Left hip biopsy	1/5	N	Y (11)	Levofloxacin	N	Colonization or contamination
7	Orthopedic surgery	08/08/2019 to 29/08/2019 (21)	90	F	27	Knee prosthesis infection	19/08/2019	Right knee biopsy	5/5	N	Y (10)	Amoxicillin PTZ Levofloxacin Rifampicin	Y: doxycycline	Infection
8	Orthopedic surgery	17/03/2020 to 31/03/2020 (14)	62	M	29.4	Intercostal chondrosarcoma	24/03/2020	Coastal biopsy	5/5	N	N	N	Y: dalbavancin	Infection
9	Orthopedic surgery	27/02/2020 to 07/04/2020 (40)	59	F	23.4	Hip prosthesis infection early recurrence	15/04/2020	Right hip biopsy	1/5	N	Y (7)	PTZ TMP-SMX	Y: doxycycline	Colonization or contamination
10	Orthopedic surgery	10/05/2020 to 28/05/2020 (18)	45	M	20.6	3 months post-sacrotomy urinary sepsis	25/05/2020	Perineal abscess drainage	1/2 (Enriched broth culture only)	N	Y (2 + 7)	AMC PTZ Gentamicin Meropenem Cefotaxime Ciprofloxacin	N	Colonization or contamination
11	Surgical ICU	01/06/2020 to 04/07/2020 (33)	50	F	62.7	Mediastinitis and septic shock	11/06/2020	Mediastinal and pre-sternal drainage	4/4	N	N	AMC Ceftriaxone Metronidazole Gentamicin	Y: vancomycin	Infection
12	Orthopedic surgery	28/07/2020 to 02/09/2020 (36)	64	M	23.3	Ankle fracture	15/08/2020	Synovial fluid	1/3	N	Y (11)	AMC Gentamicin PTZ Levofloxacin Moxifloxacin	N	Colonization or contamination
13	Surgical ICU	19/09/2020 to 13/11/2020 (55)	73	M	35	Liver transplant	23/09/2020	Blood	1/6 bottle	N	Y (5)	PTZ Tobramycin Cloxacillin Vancomycin Gentamycin	Y: vancomycin	Contamination
14	Orthopedic surgery	16/09/2020 to 24/09/2020 (8)	70	M	32.7	Acute sepsis, one-stage knee prosthesis exchange	17/09/2020	Biopsy	4/5	Y: 1/5 MRSE (15/12/2019)	Y (4)	TMP-SMX Ciprofloxacin PTZ	Y: dalbavancin then doxycycline	Infection
15	Orthopedic surgery	02/10/2020 to 03/11/2020 (32)	73	F	26	Two-stage hip prosthesis exchange	16/10/2020	Right hip biopsy	5/5	N	Y (5)	PTZ Ciprofloxacin	Y: vancomycin then dalbavancin	Infection
16	Orthopedic surgery	16/12/2020 to 02/02/2021 (48)	19	M	34.6	Femoral fracture	22/01/2021	Hip biopsy	1/3	N	Y (5)	PTZ Cefepime Moxifloxacin Meropenem Levofloxacin	Y: teicoplanin then doxycycline	Colonization or contamination
17	Orthopedic surgery	08/12/2020 to 19/02/21 (73)	82	F	33.8	One-stage knee prosthesis exchange	28/01/2021	Right hip biopsy	3/5	Y: 4/5 MRSE (21/12/2020)	Y (14) Tedizolid (4)	PTZ	Y: Daptomycin and doxycycline	Infection
18	Surgical ICU	01/02/2021 to 12/02/2021 (12)	68	M	34.4	Cardiogenic refractory shock	05/02/2021	Blood	1/4 bottle	N	Y (9)	AMC Daptomycin PTZ	Y: vancomycin	Contamination
19	Orthopedic surgery	09/07/2021 to 13/08/2021 (35)	88	M	28	Hip prosthesis removal	04/08/2021	Left hip biopsy	3/4	N	Y (6 + 10)	PTZ Cefepime Rifampicin ciprofloxacin	N (palliative care)	Infection
20	Surgical ICU	08/10/2021 to 18/11/2021 (41)	68	M	27.9	Liver transplant	16/10/2021	Peritoneal fluid	1/4	N	N	PTZ Meropenem Vancomycin	Y: vancomycin	Infection or colonization

AMC, amoxicillin-clavulanic acid; PTZ, piperacillin-tazobactam; TMP-SMX, trimethoprim-sulfamethoxazole; MSSE, Methicillin-susceptible *S. epidermidis*; MRSE, Methicillin-resistant *S. epidermidis*; ND, No Data.

**Figure 2:**
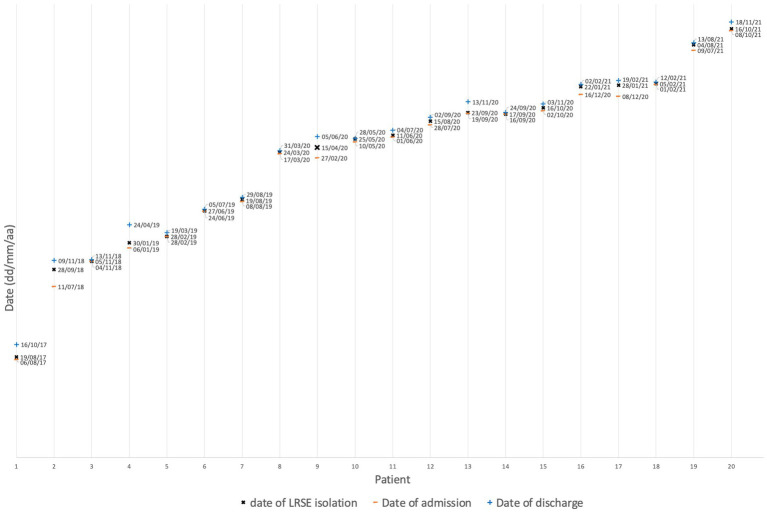
Dates of patient hospitalization and isolation of LRSE strains.

Fourteen of 20 patients were exposed to linezolid in the 3 months prior to the onset of LRSE, with exposure ranging from five to 16 days (Table 1). The patients’ hospitalization date and LRSE isolation date are shown in Figure 2. No patients were found to have shared a double room or stayed successively in the same room within a department.

### 3.2 Linezolid usage data

**Figure 3:**
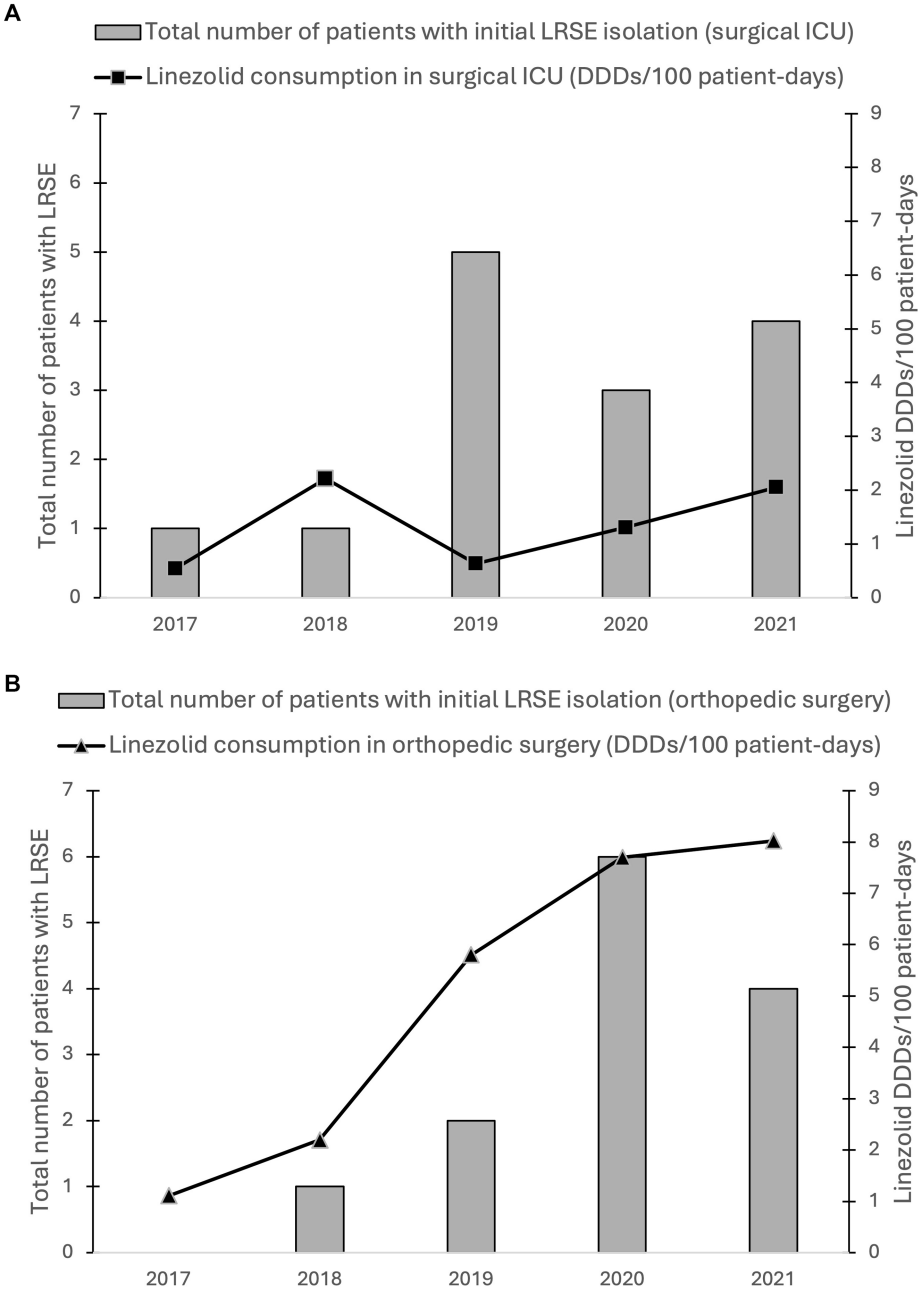
Annual linezolid consumption data from **(A)** the surgical ICU and **(B)** the orthopedic surgery from 2017 to 2021 versus the total number of LRSE isolates, including those that were not preserved. Linezolid use was calculated in DDDs/100 patient-days. Doses of 1,200 mg/day were considered as 1 DDD. Bars indicate the number of patients with initial detection of LRSE in the respective year in the unit.

We reviewed linezolid consumption in surgical ICU and orthopedic surgery (Figure 3). In relation to hospital-wide linezolid use at our institution, the surgical ICU and orthopedic surgery accounted for 1.5 and 16.7% of the total number of linezolid dosage units dispensed in 2017, 2.8 and 15.6% in 2018, 0.63 and 30.7% in 2019, 1.2 and 30.0% in 2020, and 1.7 and 28.7% in 2021, respectively.

### 3.3 Clonality of LRSE isolates: PFGE and MLST analysis

Twenty LRSE isolates were genotyped and shared the same PFGE pulsotype (A), with the exception of one (called pulsotype B) obtained from patient 11 (Figure 1). The three LSSE were not genetically related to each other or to the LRSE clone, demonstrating the genomic diversity among susceptible strains (Figure 1). The PFGE results were confirmed by MLST typing, which showed that all LRSE isolates with pulsotype A belonged to ST2. The pulsotype B strain belonged to ST57. The LSSE strains were ST21, ST87, and ST470 (Figure 1).

### 3.4 Correlation between IR-Biotyper^®^ dendrogram and PFGE

The ability of the IRBT to assess the degree of genomic relatedness between isolates using a dendrogram generated by the instrument was evaluated and compared to PFGE, considered the gold standard in this study. IRBT clustering showed a clonal distribution of the population similar to that obtained by PFGE (Figure 1). Due to colony dissociation, one strain was tested twice. The PFGE profiles of both isolates were identical, whereas IRBT surprisingly identified two different spectra. Eight IR-spectra were identified among the isolates: IRBT detected clonality of 16 of the 19 ST2 LRSE strains of pulsotype A and distinguished the non-ST2 LRSE strain of pulsotype B and the three LSSE strains from the clonal strain. However, the IRBT assigned different IR-spectra to the Pulsotype A LRSE strains of Patient 1 and Patient 6 (Figure 1). The discriminatory power of IRBT was calculated using Simpson’s index of diversity. It assesses the reliability of IRBT in distinguishing between unrelated strains (Hunter and Gaston, 1988). The concordance of clusters of related strains identified by PFGE and IRBT was determined by calculating the adjusted Wallace coefficient (Severiano et al., 2020). These results are presented in Table 2, together with the sensitivity, specificity, positive predictive value and negative predictive value of IRBT.

**Table 2:** Discriminatory power, concordance between IRBT and PFGE and performance of IRBT.

Simpson’s index of diversity (95% CI)	Adjusted Wallace coefficient (95% CI)	Sensitivity	Specificity	Positive predictive value	Negative predictive value
0.565 (0.325–0.806)	0.472 (0.002–0.943)	0.842	1.00	1.00	0.714

CI, confidence interval.

### 3.5 Antimicrobial susceptibility and determinants of methicillin and linezolid resistance

LRSE isolates were resistant to methicillin, gentamicin, ofloxacin, erythromycin, clindamycin, rifampicin, and cotrimoxazole. No resistance to vancomycin or teicoplanin was observed. LRSE showed a high level of resistance to linezolid, with MICs ≥ 256 mg/L, and to tedizolid (MICs > 32 mg/L), except for the pulsotype B LRSE strain. Surprisingly, this latter was susceptible to tedizolid (MIC = 0.19 mg/L) (Table 3). Most LRSE isolates were susceptible to delafloxacin (18/20), but resistant to ceftaroline (13/20), and ceftobiprole (19/20). Methicillin resistance was due to the presence of the *mecA* gene. Analysis of the 23S rRNA fragment performed on 17 of the 20 LRSE strains showed the G2576T mutation (regardless of pulsotype), as previously described in clinical isolates of LRSE (Bouiller et al., 2020; Coustillères et al., 2023; Treviño et al., 2009). No transferable oxazolidinone resistance genes, including the *cfr*, *optrA* and *poxtA* genes, were acquired by any of these 17 strains.

**Table 3:** Antimicrobial susceptibility results of 20 LRSE and three LSSE strains, and characterization of the resistance determinants.

MICs (mg/L)													Mutation
Patient	Strain	Methi-R (Y/N)	VAN	TEC	TIG	DLF	DLB	LNZ	TDZ	DPT	CFT	CTB	23S rRNA
1	LRSE	Y	2	2	0.25	0.032	0.060	>256	>32	0.50	0.50	3	G2576T
2	LRSE	Y	1	1	0.25	0.032	0.064	>256	>32	0.50	0.50	2	ND
3	LRSE	Y	1	2	0.50	0.094	0.094	>256	>32	0.50	0.75	4	G2576T
	LSSE	N	1	0.25	0.094	0.004	0.016	0.75	0.025	0.50	0.094	0.5	WT
4	LRSE	Y	1	1	0.094	0.032	0.047	>256	>32	0.38	0.75	3	G2576T
5	LRSE	Y	1	1	0.19	0.064	0.064	>256	>32	0.75	2	4	G2576T
6	LRSE	Y	1	1	0.50	0.38	0.94	>256	16	0.75	0.75	4	ND
7	LRSE	Y	1	0.50	0.50	0.032	0.094	>256	>32	0.75	2	4	G2576T
8	LRSE	Y	1	0.50	0.38	0.047	0.060	>256	>32	0.50	2	4	G2576T
9	LRSE	Y	1	0.50	0.19	0.19	0.064	>256	>32	0.25	2	4	G2576T
10	LRSE	Y	1	0.50	0.064	0.032	0.064	>256	>32	0.38	1.5	4	G2576T
11	LRSE	Y	0.50	0.50	0.094	0.047	0.047	256	0.19	0.19	0.38	1	G2576T
12	LRSE	Y	1	0.50	0.38	0.064	0.064	>256	>32	0.38	1.5	4	G2576T
13	LRSE	Y	2	2	0.25	0.032	0.094	>256	>32	0.38	2	4	G2576T
14	LRSE	Y	1	2	0.25	0.094	0.094	>256	>32	0.25	0.75	4	G2576T
	LSSE	Y	0.50	0.50	0.047	0.023	0.016	1	0.008	0.125	0.094	0.075	WT
15	LRSE	Y	1	0.50	0.38	0.064	0.064	>256	>32	0.50	1.5	4	G2576T
16	LRSE	Y	1	0.50	0.25	0.023	0.064	>256	>32	0.25	2	4	ND
17	LRSE	Y	1	0.50	0.38	0.064	0.094	>256	>32	0.75	1.5	3	G2576T
	LSSE	Y	2	2	0.047	0.064	0.032	0.75	0.094	0.25	0.5	0.5	WT
18	LRSE	Y	1	0.50	0.38	0.032	0.064	>256	>32	0.75	2	4	G2576T
19	LRSE	Y	1	0.50	0.38	0.094	0.060	>256	>32	0.50	2	4	G2576T
20	LRSE	Y	1	0.50	0.25	0.032	0.060	>256	>32	0.50	2	4	G2576T

Methi-R, methicillin-resistant; VAN, vancomycin; TEC, teicoplanin; TIG, tigecycline; DLF, delafloxacin; DLB, dalbavancin; LNZ, linezolid; TDZ, tedizolid; DPT, daptomycin; CFT, ceftaroline; CTB, ceftobiprole; ND, No Data; WT, Wild-Type.

## 4 Discussion

In our study, we phenotypically and genotypically characterized the LRSE strains involved in an outbreak at the Tours University Hospital between 2017 and 2021. All LRSE strains, even the non-clonal one, showed high levels of resistance to linezolid, with MICs ≥ 256 mg/L. All 17 LRSE strains analyzed had the G2576T mutation in domain V of the 23S rRNA gene, which appears to be most common in clinical settings (Pillai et al., 2002). None of the 17 strains analyzed carried the *cfr* gene, limiting concerns about plasmid-mediated resistance spreading to more pathogenic *Staphylococcus* species such as *S. aureus*.

PFGE showed that all but one of the LRSE strains tested were genetically related. They belonged to the ST2 according to MLST, which appears consistent with previous studies reporting LRSE clonal occurrence of ST2 (Bouiller et al., 2020; Coustillères et al., 2023; Kosecka-Strojek et al., 2020). However, ST2 appears to be a common lineage in *S. epidermidis*, regardless of linezolid susceptibility (Martínez-Santos et al., 2022). Although PFGE is still a routinely used tool in nosocomial outbreak investigations, it would have been interesting for this study to perform whole genome sequencing (WGS), which provides greater resolution than any other microbial strain typing method.

We used IRBT to analyze the outbreak after PFGE typing. It was mostly congruent with the PFGE results: IRBT showed 87% congruence with PFGE analysis. One limitation of our study, however, is that because IRBT was performed retrospectively, it could have introduced a bias into our analysis. Also, IRBT discriminated two deposits of the same strain as two different IR-types. Because our IRBT experiment was only performed once, we assumed this could have been caused by technical factors. Random error or a contamination could also not be excluded. Moreover, our study included only a small number and low diversity of strains tested, so further studies are needed to assess the optimal analytical parameters to validate the technique for routine epidemiological use. Rakovitsky et al. showed that performing a dozen replicates in a single run, instead of three or four, can optimize the generated cut-off value, thus limiting the under- or over-sensitivity of the IRBT analysis (Rakovitsky et al., 2020). To date, our study is the first one to evaluate IRBT for *S. epidermidis*, but previous studies have shown promising results for the typing of other microorganisms, including for clinical outbreak investigation purposes (Hong et al., 2022; Hu et al., 2020; Pascale et al., 2022; Rakovitsky et al., 2020). IRBT is less expensive than PFGE, with a cost of 20 Euros per sample analyzed in triplicate, although it requires the purchase of an automated system. In comparison, PFGE costs 50 Euros per sample. IRBT is technically easy to use, and a sample can be prepared in about 20 min, compared to 4–5 h for PFGE, which requires skilled technicians. Thus, due to its short turnaround time, IRBT appears to be a promising tool for outbreak investigation. Results can be obtained in a matter of hours (compared to several days for PFGE), allowing infection control measures to be implemented quickly to contain the outbreak.

Regarding the emergence of the LRSE epidemic clone, three patients (3, 14, 17) harbored linezolid-susceptible *S. epidermidis* (LSSE) strains a few months before isolation of the clone, with, respectively, 1/5, 1/5, and 4/5 positive osteoarticular intraoperative specimens. As the LSSE strains belonged to a different clone from the LRSE strain, this may argue against the selection of linezolid resistance mutations in pre-existing LSSE strains.

Most patients (at least 14 out of 20) had prior exposure to linezolid in the 3 months prior to isolation of the LRSE clone. The orthopedic surgery department and the surgical ICU were at the center of the outbreak. Orthopedic surgery was the biggest user of linezolid, with consumption accounting for almost a third of the total doses dispensed by Tours University Hospital in 2019, 2020, and 2021. Previous studies have mainly highlighted the direct link with the consumption of linezolid in healthcare establishments. In fact, selective antimicrobial pressure may promote the survival and spread of LRSE strains by suppressing patients’ susceptible microbiota, allowing resistant strains to predominate (Huber et al., 2021; Weßels et al., 2018). Thus, limiting the prescription of oxazolidinones could help to limit this incidence (Dortet et al., 2018).

However, an Austrian institution reported an increase in the isolation of LRSE strains, with a peak of 84 isolates in 2018, despite the reduction in linezolid consumption compared with 2012 and 2013 (Huber et al., 2021). The authors reported that 47 of the 347 patients had no history of linezolid administration in the year prior to isolation of LRSE strains.

The development of linezolid resistance does not appear to be triggered solely by the use of linezolid. Indeed, the number of LRSE isolates appears to be associated with linezolid use data in orthopedic surgery, but not in the surgical ICU. The total number of LRSE strains was similar in both departments, whereas linezolid consumption was lower in the surgical ICU.

In our study, 5 out of 20 patients did not receive linezolid prior to isolation of the LRSE strain, suggesting the hypothesis of a reservoir and hand transmission of the clone by healthcare workers.

In addition, more than 40% of LRSE clonal strains were considered colonization or contamination. Indeed, the clinical histories of two patients (13 and 18) showed that the LRSE strain isolated in their blood cultures was a contaminant, suggesting (i) skin colonization of the patients with this strain and/or (ii) hand-carriage of this strain by healthcare workers. These two patients were hospitalized in the same department, but never shared the same room. A year and a half separated the two stays. Given that carriage of the strain by healthcare staff would probably be more transient, we cannot rule out the possibility that contaminated surfaces, such as computer keyboards, acted as a reservoir for the LRSE strain.

The retrospective nature of the study did not allow us to screen patients and healthcare providers in these units for carriage, nor to sample the environment, thus limiting exploration of the chain of transmission. It is likely that infection with the resistant strain was linked to surface contamination, followed by hand-transmission by healthcare workers, leading to colonization of patients’ skin. Since most of the patients in our study had previously received linezolid, this antibiotic may have suppressed the susceptible skin microbiota, allowing the LRSE clone to take advantage of this niche.

Kelly et al. (2008) reported an outbreak of a LRSE clonal strain in 16 ICU patients: the epidemic clonal strain was found in the ICU environment and surfaces and may therefore be a reservoir. None of the 58 ICU healthcare workers screened for nasal carriage were carriers of the epidemic strain, but transient skin carriage may have occurred during the early stages of the outbreak. Other studies have reported transmission of *S. epidermidis* by healthcare workers through hand contact (Christensen et al., 1982; Kotilainen et al., 1990; Simpson et al., 1986). Although 6 of 16 patients did not receive linezolid, the authors reported a significant increase in linezolid use prior to the LRSE clonal outbreak. Restriction of linezolid use and reinforcement of contact precautions eradicated the outbreak (Kelly et al., 2008). A Spanish study also reported an outbreak of linezolid resistant *S. aureus*. Again, none of the healthcare providers carried the epidemic clone, but it was recovered from environmental surfaces (Sánchez García et al., 2010).

To maintain the efficacy of linezolid, it is important to carefully manage its use. The increase in linezolid resistance in *S. epidermidis* should temper its probabilistic use in infections commonly caused by *Staphylococci*, such as catheter-associated bloodstream infections.

The multidrug-resistant ST2 LRSE clone was able to persist and spread within the same hospital for 4 years and, to our knowledge, may continue to spread today. This is why the emergence of resistant clones should be detected at an early stage so that appropriate measures may be taken to control their spread, especially since CoNS are known to be a reservoir of resistance genes (Côrtes et al., 2022).

## Data Availability

The raw data supporting the conclusions of this article will be made available by the authors, without undue reservation.

## Footnotes

^1^ https://pubmlst.org/organisms/staphylococcus-epidermidis

## References

[ref1] Bouiller K. Ilic D. Wicky P. H. Cholley P. Chirouze C. Bertrand X. (2020). Spread of clonal linezolid-resistant *Staphylococcus epidermidis* in an intensive care unit associated with linezolid exposure. Eur. J. Clin. Microbiol. Infect. Dis. 39, 1271–1277. doi: 10.1007/s10096-020-03842-7 32060752

[ref2] Brenciani A. Morroni G. Schwarz S. Giovanetti E. (2022). Oxazolidinones: mechanisms of resistance and mobile genetic elements involved. J. Antimicrob. Chemother. 77, 2596–2621. doi: 10.1093/jac/dkac263, PMID: 35989417

[ref3] Christensen G. D. Bisno A. L. Parisi J. T. McLaughlin B. Hester M. G. Luther R. W. (1982). Nosocomial septicemia due to multiply antibiotic-resistant *Staphylococcus epidermidis*. Ann. Intern. Med. 96, 1–10. doi: 10.7326/0003-4819-96-1-1, PMID: 7053681

[ref4] Côrtes M. F. André C. Martins Simões P. Corvec S. Caillon J. Tristan A.. (2022). Persistence of a multidrug-resistant worldwide-disseminated methicillin-resistant *Staphylococcus epidermidis* clone harbouring the *cfr* linezolid resistance gene in a French hospital with evidence of interspecies transfer to several *Staphylococcus aureus* lineages. J. Antimicrob. Chemother. 77, 1838–1846. doi: 10.1093/jac/dkac119, PMID: 35425984

[ref5] Coustillères F. Renault V. Corvec S. Dupieux C. Simões P. M. Lartigue M. F.. (2023). Clinical, bacteriological, and genetic characterization of bone and joint infections involving linezolid-resistant *Staphylococcus epidermidis*: a retrospective multicenter study in French reference centers. Microbiol. Spectr. 11:e0419022. doi: 10.1128/spectrum.04190-22 37133395 PMC10269892

[ref6] Dortet L. Glaser P. Kassis-Chikhani N. Girlich D. Ichai P. Boudon M.. (2018). Long-lasting successful dissemination of resistance to oxazolidinones in MDR *Staphylococcus epidermidis* clinical isolates in a tertiary care hospital in France. J. Antimicrob. Chemother. 73, 41–51. doi: 10.1093/jac/dkx370, PMID: 29092052 PMC5890688

[ref7] Flamm R. K. Mendes R. E. Hogan P. A. Streit J. M. Ross J. E. Jones R. N. (2016). Linezolid surveillance results for the United States (LEADER surveillance program 2014). Antimicrob. Agents Chemother. 60, 2273–2280. doi: 10.1128/AAC.02803-15, PMID: 26833165 PMC4808230

[ref8] Hong J. S. Kim D. Jeong S. H. (2022). Performance evaluation of the IR Biotyper® system for clinical microbiology: application for detection of *Staphylococcus aureus* sequence type 8 strains. Antibiotics 11:909. doi: 10.3390/antibiotics11070909, PMID: 35884163 PMC9311605

[ref9] Hu Y. Zhou H. Lu J. Sun Q. Liu C. Zeng Y.. (2020). Evaluation of the IR Biotyper for *Klebsiella pneumoniae* typing and its potentials in hospital hygiene management. Microb. Biotechnol. 14, 1343–1352. doi: 10.1111/1751-7915.13709, PMID: 33205912 PMC8313285

[ref10] Huber S. Knoll M. A. Berktold M. Würzner R. Brindlmayer A. Weber V.. (2021). Genomic and phenotypic analysis of linezolid-resistant *Staphylococcus epidermidis* in a tertiary Hospital in Innsbruck, Austria. Microorganisms 9:1023. doi: 10.3390/microorganisms9051023, PMID: 34068744 PMC8150687

[ref11] Hunter P. R. Gaston M. A. (1988). Numerical index of the discriminatory ability of typing systems: an application of Simpson’s index of diversity. J. Clin. Microbiol. 26, 2465–2466. doi: 10.1128/jcm.26.11.2465-2466.1988, PMID: 3069867 PMC266921

[ref12] Kelly S. Collins J. Maguire M. Gowing C. Flanagan M. Donnelly M.. (2008). An outbreak of colonization with linezolid-resistant *Staphylococcus epidermidis* in an intensive therapy unit. J. Antimicrob. Chemother. 61, 901–907. doi: 10.1093/jac/dkn043 18272512

[ref13] Kosecka-Strojek M. Sadowy E. Gawryszewska I. Klepacka J. Tomasik T. Michalik M.. (2020). Emergence of linezolid-resistant *Staphylococcus epidermidis* in the tertiary children’s hospital in Cracow, Poland. Eur. J. Clin. Microbiol. Infect. Dis. 39, 1717–1725. doi: 10.1007/s10096-020-03893-w, PMID: 32350737 PMC7427702

[ref14] Kotilainen P. Nikoskelainen J. Huovinen P. (1990). Emergence of ciprofloxacin-resistant coagulase-negative staphylococcal skin flora in immunocompromised patients receiving ciprofloxacin. J. Infect. Dis. 161, 41–44. doi: 10.1093/infdis/161.1.41, PMID: 2295857

[ref15] Liakopoulos A. Spiliopoulou I. Damani A. Kanellopoulou M. Schoina S. Papafragas E.. (2010). Dissemination of two international linezolid-resistant *Staphylococcus epidermidis* clones in Greek hospitals. J. Antimicrob. Chemother. 65, 1070–1071. doi: 10.1093/jac/dkq065, PMID: 20207718

[ref16] Long K. S. Vester B. (2012). Resistance to linezolid caused by modifications at its binding site on the ribosome. Antimicrob. Agents Chemother. 56, 603–612. doi: 10.1128/AAC.05702-11, PMID: 22143525 PMC3264260

[ref17] Martínez-Santos V. I. Torres-Añorve D. A. Echániz-Aviles G. Parra-Rojas I. Ramírez-Peralta A. Castro-Alarcón N. (2022). Characterization of *Staphylococcus epidermidis* clinical isolates from hospitalized patients with bloodstream infection obtained in two time periods. Peer J 10:e14030. doi: 10.7717/peerj.14030, PMID: 36213498 PMC9541613

[ref18] Mihaila L. Defrance G. Levesque E. Ichai P. Garnier F. Derouin V.. (2012). A dual outbreak of bloodstream infections with linezolid-resistant Staphylococcus epidermidis and *Staphylococcus pettenkoferi* in a liver intensive care unit. Int. J. Antimicrob. Agents 40, 472–474. doi: 10.1016/j.ijantimicag.2012.06.014, PMID: 22883415

[ref19] Neoh H. Tan X.-E. Sapri H. F. Tan T. L. (2019). Pulsed-field gel electrophoresis (PFGE): a review of the “gold standard” for bacteria typing and current alternatives. Infect. Genet. Evol. 74:103935. doi: 10.1016/j.meegid.2019.103935, PMID: 31233781

[ref20] Pascale M. R. Bisognin F. Mazzotta M. Girolamini L. Marino F. Dal Monte P.. (2022). Use of Fourier-transform infrared spectroscopy with IR Biotyper® system for *Legionella pneumophila* serogroups identification. Front. Microbiol. 13:866426. doi: 10.3389/fmicb.2022.866426, PMID: 35558114 PMC9090449

[ref21] Pillai S. K. Sakoulas G. Wennersten C. Eliopoulos G. M. Moellering R. C. Ferraro M. J.. (2002). Linezolid resistance in *Staphylococcus aureus*: characterization and stability of resistant phenotype. J. Infect. Dis. 186, 1603–1607. doi: 10.1086/345368, PMID: 12447736

[ref22] Rakovitsky N. Frenk S. Kon H. Schwartz D. Temkin E. Solter E.. (2020). Fourier transform infrared spectroscopy is a new option for outbreak investigation: a retrospective analysis of an extended-Spectrum-Beta-lactamase-producing *Klebsiella pneumoniae* outbreak in a neonatal intensive care unit. J. Clin. Microbiol. 58, e00098–e00020. doi: 10.1128/JCM.00098-20, PMID: 32161093 PMC7180251

[ref23] Sánchez García M. De la Torre M. A. Morales G. Peláez B. Tolón M. J. Domingo S.. (2010). Clinical outbreak of linezolid-resistant *Staphylococcus aureus* in an intensive care unit. JAMA 303, 2260–2264. doi: 10.1001/jama.2010.757, PMID: 20530779

[ref24] Seral C. Sáenz Y. Algarate S. Duran E. Luque P. Torres C.. (2011). Nosocomial outbreak of methicillin- and linezolid-resistant *Staphylococcus epidermidis* associated with catheter-related infections in intensive care unit patients. Int. J. Med. Microbiol. 301, 354–358. doi: 10.1016/j.ijmm.2010.11.001, PMID: 21236728

[ref25] Severiano A. Pinto F. R. Ramirez M. Carriço J. A. (2020). Adjusted Wallace coefficient as a measure of congruence between typing methods. J. Clin. Microbiol. 49, 3997–4000. doi: 10.1128/jcm.00624-11, PMID: 21918028 PMC3209087

[ref26] Simpson R. A. Spencer A. F. Speller D. C. Marples R. R. (1986). Colonization by gentamicin-resistant *Staphylococcus epidermidis* in a special care baby unit. J. Hosp. Infect. 7, 108–120. doi: 10.1016/0195-6701(86)90053-8, PMID: 2871073

[ref27] Thomas J. C. Vargas M. R. Miragaia M. Peacock S. J. Archer G. L. Enright M. C. (2007). Improved multilocus sequence typing scheme for *Staphylococcus epidermidis*. J. Clin. Microbiol. 45, 616–619. doi: 10.1128/JCM.01934-06, PMID: 17151213 PMC1829011

[ref28] Treviño M. Martínez-Lamas L. Romero-Jung P. A. Giráldez J. M. Alvarez-Escudero J. Regueiro B. J. (2009). Endemic linezolid-resistant *Staphylococcus epidermidis* in a critical care unit. Eur. J. Clin. Microbiol. Infect. Dis. 28, 527–533. doi: 10.1007/s10096-008-0657-5, PMID: 18985396

[ref29] Weßels C. Strommenger B. Klare I. Bender J. Messler S. Mattner F.. (2018). Emergence and control of linezolid-resistant *Staphylococcus epidermidis* in an ICU of a German hospital. J. Antimicrob. Chemother. 73, 1185–1193. doi: 10.1093/jac/dky010, PMID: 29438544

