# Supplementary material for: Investigation of a linezolid-resistant Staphylococcus epidermidis outbreak in a French hospital: phenotypic, genotypic, and clinical characterization
Source: Front Microbiol. 2024 Sep 11;15:1455945. doi: 10.3389/fmicb.2024.1455945 (PMC11422107; doi:10.3389/fmicb.2024.1455945)
Supplement: Supplementary file 1 [file Table_1.DOCX]

Supplementary Material

Investigation of a linezolid-resistant Staphylococcus epidermidis outbreak in a French hospital: phenotypic, genotypic, and clinical characterization

Nadège Lépine^1,2^*, José Bras-Cachinho^1^, Eva Couratin^3^, Coralie Lemaire^1,2^, Laura Chaufour^1^, Armelle Junchat^3^, Marie-Frédérique Lartigue^1,2^*

*** Correspondence:**

Corresponding Authors: nadege.lepine@etu.univ-tours.fr, lartigue@univ-tours.fr

# Supplementary Figures and Tables

## Supplementary Table

**Supplementary Table 1.** Primers used for MLST

| **Gene and function** | **Primer** | **Sequence (5’- 3’)** | **Size of amplicon used for allele assignment (pb)** |
| --- | --- | --- | --- |
| Carbamate Kinase (arcC) | arcC-F | TGTGATGAGCACGCTACCGTTAG | 465 |
|  | arcC-R | TCCAAGTAAACCCATCGGTCTG |  |
| Shikimate dehydrogenase (aroE) | aroE-F | CATTGGATTACCTCTTTGTTCAGC | 420 |
|  | aroE-R | CAAGCGAAATCTGTTGGGG |  |
| ABC transporter (gtr) | gtr-F | CAGCCAATTCTTTTATGACTTTT | 438 |
|  | gtr-R | GTGATTAAAGGTATTGATTTGAAT |  |
| DNA mismatch repair protein (mutS) | mutS-F | GATATAAGAATAAGGGTTGTGAA | 412 |
|  | mutS-R | GTAATCGTCTCAGTTATCATGTT |  |
| Pyrimidine operon regulatory protein (pyrR) | pyrR-F | GTTACTAATACTTTTGCTGTGTTT | 428 |
|  | pyrR-R | GTAGAATGTAAAGAGACTAAAATGAA |  |
| Triosephosphate isomerase (tpiA) | tpiA-F | ATCCAATTAGACGCTTTAGTAAC | 424 |
|  | tpiA-R | TTAATGATGCGCCACCTACA |  |
| Acetyl coenzyme A acetyltransferase (yqiL) | yqiL-F | CACGCATAGTATTAGCTGAAG | 416 |
|  | yqil-R | CTAATGCCTTCATCTTGAGAAATAA |  |
